# Identification of salvianolic acid A as an ADP receptor-selective and Gq/IP_3_ pathway-mediated anti-platelet component in Qishen Yiqi

**DOI:** 10.1186/s13020-025-01188-w

**Published:** 2025-10-06

**Authors:** Wenli Dang, Liping Chen, Qinhua Shang, Tiechan Zhao, Lianying Chang, Taiyi Wang, Ming Lyu, Xiaoxuan Tian, Hao Guo, Shuang He, Jingyang Hu, Peng Zhang, Yan Zhu

**Affiliations:** 1 State Key Laboratory of Component-Based Chinese Medicine, Tianjin University of Traditional Chinese Medicine, Tianjin, 301617 China; 2 Tianjin Haihe Laboratory of Modern Chinese Medicine, Tianjin University of Traditional Chinese Medicine, Tianjin, 301617 China; 3 Research Institute of Acupuncture and Moxibustion, Shandong University of Traditional Chinese Medicine, Jinan, 250355 China

**Keywords:** Antiplatelet therapy, Platelet activation, Thrombosis, G-protein coupled receptors, Salvianolic acid A, Qishen Yiqi dripping pill

## Abstract

**Background:**

Antiplatelet therapy is crucial for preventing and treating cardio-cerebrovascular diseases. However, adverse events related to thrombosis or bleeding have been reported in instances of treatment with glycoprotein IIb/IIIa antagonists. It is anticipated that developing new selective platelet inhibitors with high anti-thrombotic efficiency and minimal hemorrhagic side effects is feasible. Qishen Yiqi Dripping Pill (QSYQ), an approved drug for ischemic heart disease, was studied for its anti-thrombotic effects.

**Methods and results:**

Employing a microplate-based platelet aggregation assay, we systematically evaluated QSYQ and its medicinal components, chemical fractions, and compounds from the active fractions, identifying Salvianolic acid A (SAA) as one of the major active components for platelet inhibition. Our findings revealed that SAA decreased platelet [Ca^2+^]_i_ via the G_q_/IP_3_ pathway without affecting cAMP levels. Furthermore, 20 mg/kg SAA reduced thrombus formation in a ferric chloride (FeCl_3_)-induced thrombotic model in vivo, suggesting the pharmacological significance of SAA in QSYQ.

**Conclusion:**

This study identified SAA as one of the pharmacologically active anti-platelet components in QSYQ and revealed that its mechanism of action operates via the G_q_/IP_3_ signaling pathway.

**Graphical Abstract:**

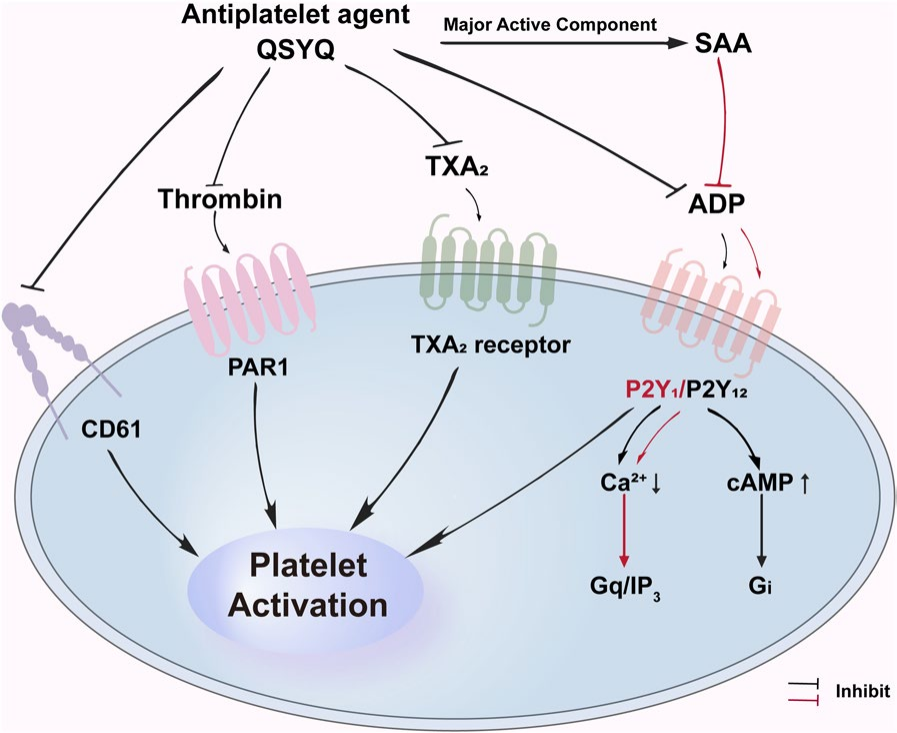

**Supplementary Information:**

The online version contains supplementary material available at 10.1186/s13020-025-01188-w.

## Introduction

Thrombosis represents a major pathogenic mechanism underlying diverse cardiovascular diseases and constitutes a leading global cause of mortality [1]. Platelets are central to physiological hemostasis through processes including morphological alteration, adhesion, and aggregation. However, dysregulation of these processes, characterized by excessive platelet activation, pathological adhesion, and abnormal aggregation, predisposes to thrombotic disorders [2–4]. Crucially, platelet activation and aggregation drive thrombus formation and vessel occlusion at the site of coronary arterial plaque rupture [5, 6]. G protein-coupled receptors (GPCRs) on the platelet membrane, including receptors for adenosine diphosphate (ADP), thrombin, and thromboxane A_2_ (TXA_2_), play a pivotal role in atherothrombosis [7, 8]. Consequently, antiplatelet therapies targeting key platelet activation pathways are crucial for managing cardiovascular disease [9].

Current antiplatelet strategies inhibit specific targets, including thromboxane A2 (TXA_2_) synthesis, ADP signaling, integrin αIIbβ3 (GPIIb/IIIa), thrombin-mediated platelet activation via the protease-activated receptor 1 (PAR1), and phosphodiesterase activity [10]. Despite the development of numerous antiplatelet or antithrombotic agents aimed at improving the prevention of arterial or venous thrombosis, current single-component antiplatelet drugs have limitations, including aspirin resistance, increased bleeding risk [11], and clopidogrel-induced hypo-responsiveness [12]. Given the persistently high incidence of thrombotic events among patients receiving current single antiplatelet therapies, combination therapies have become a prevalent approach in antiplatelet treatment strategies.

Compound Chinese herbal medicine, a natural multiplex medicine, has been a rich source of drug candidates for cardiovascular diseases [13]. A group of herbs with “blood-activating” properties, “Dan Shen” dripping pill, composed of Salvia miltiorrhiza, Notoginseng, and Borneol, significantly inhibited platelet aggregation, platelet-leucocyte conjugate formation, and leucocyte activation [14]. Qishen Yiqi Dripping Pills (QSYQ), composed of *Radix Astragali*, *Salvia miltiorrhiza*, *Notoginseng*, and *Dalbergia odorifera*, have been used for treating ischemic heart diseases, such as chronic cardiac insufficiency, congestive heart failure, angina pectoris, and coronary heart disease in the clinic [15, 16]. According to traditional Chinese medicine theory, this drug has the effect of “tonifying Qi”, activating blood circulation, and alleviating pain in patients suffering from coronary disease and angina, as well as improving ventricular remodeling. A randomized, double-blind clinical trial involving over 3500 patients showed that QSYQ was as effective as Aspirin in the secondary prevention of myocardial infarction [17]. Results from a randomized, double-blind, multicenter placebo-controlled study enrolled 640 patients showed that treatment with QSYQ for 6 months in addition to standard therapy improved exercise tolerance of ischemic heart failure (IHF) patients [18]. Most recently, a secondary analysis of data from a multicenter, prospective cohort study involving 1225 patients with IHF from 84 centers showed that QSYQ can further improve EF classification, reduce the occurrence rate of the composite endpoint, improve quality of life, and improve the safety profile of patients with IHF [19].

However, the pharmacological basis underlying QSYQ’s efficacy in preventing myocardial infarction remains unclear. Studies have indicated that QSYQ regulates platelet aggregation and inhibits the excessive release of β-TG in hyperlipidemic rabbits, where an elevated level of cAMP is potentially involved in this mechanism [20]. Moreover, multiple receptor-mediated signaling pathways regulate platelet activation. Therefore, this study aimed to evaluate the hypothesis that QSYQ contained antithrombotic components that exerted their effects through the inhibition of critical platelet GPCRs. We further demonstrated that SAA was one of the active antiplatelet components that inhibited ADP-induced platelet aggregation through a G_q_/IP_3_-specific signaling pathway.

## Materials and methods

Rapid platelet aggregation assays using microplate readers, cAMP measurements, [Ca^2+^]_i_ determinations, and UPLC analysis were performed according to our previously published methods [21]. Molecular docking was performed as we described previously [22]. For a detailed description of these methods, please refer to the Supplemental Information.

### Drugs and reagents

SAA and SAB were purchased from Shanghai Winherb Medical Technology Co., Ltd. (Shanghai, China). QSYQ and QSYQ full extracts (Qishenyiqi jingao, QSJG) were provided by Tasly Pharmaceutical Group Co., Ltd. (Tianjin, China). QSJG was prepared according to the ratio of Huangqi:Danshen:Sanqi:Jiangxiang oil (148.01:70.35:70.35:11.97) and diluted with ultrapure water to make a solution at a concentration of 30 mg/mL for experiments. Radix Astragali, Salvia Miltiorrhiza, and Notoginseng were provided by Tasly Pharmaceutical Group Co., Ltd. (Tianjin, China). PE anti-mouse/rat CD62P (P-selectin) Antibody, FITC anti-mouse/rat CD61 antibody were purchased from BioLegend. Aspirin was purchased from Lionco Pharmaceutical Co., Ltd. (Hainan, China). FeCl_3_ was obtained from Xiya Reagent (Shandong, China). Rhodamine 6G and 2, 2, 2-tribromoethanol were purchased from Sigma (MO, USA).

### Cell culture

The human astrocytoma1321N1 (ECACC 86030402) cell line was purchased from ECACC. Mouse brain microvascular endothelial cell line (bEnd.3) was purchased from the National Collection of Authenticated Cell Cultures (Shanghai, China). These cells were cultured in Dulbecco’s Modified Eagle’s Medium (DMEM) containing 10% fetal bovine serum and 1% penicillin/streptomycin. The U2OS β-arrestin2-RrGFP cell was a gift from PerkinElmer and maintained in McCoy's 5A (PM150710) supplemented with 10% FBS and 1% penicillin/streptomycin.

### Experimental animal

Mice (20–22 g) were obtained and maintained in a temperature-controlled room under a 12-h light/dark cycle. Mice were given water and provided with standard chow. The animal care and operational procedures followed the Animal Use Regulations of China. The experimental protocols were approved by the Institutional Animal Care and Use Committee at Tianjin International Joint Academy of Biotechnology and Medicine (Protocol number TJAB-JY-2011-001).

### Platelet and whole blood preparation

After rats were anesthetized with 3% isoflurane, whole blood was collected from the abdominal aorta in a tube containing 10% anticoagulant buffer (3 mM citric acid, 5 mM sodium citrate, and 124 mM dextrose). The platelet-rich supernatant (PRP) was collected by centrifuging citrate-anticoagulated whole blood at 200×*g* for 10 min. To avoid self-activation of platelets during isolation, 5 mM PGE1 was added. Platelets were pelleted by a second centrifugation step for 10 min at 800×*g*, resuspended in buffer A (130 mM NaCl, 10 mM sodium citrate, 9 mM NaHCO_3_, 6 mM dextrose, 0.9 mM MgCl_2_, 0.81 mM KH_2_PO_4_, 10 mM Tris, pH 7.35∼7.45), and the platelet density in the suspension was adjusted to 2 × 10^8^/mL.

### Microplate-based rapid platelet aggregation assay

100 µL of platelets were added to a 96-well microplate, and the OD values were measured by FlexStation® 3. A platelet concentration of 1 × 10^8^ platelets/mL was considered the ideal concentration for the control. We characterized changes in the platelet aggregation state by monitoring absorbance changes at 405 nm (Figure S1A).

$$\text{Aggregation }\left(\text{\%}\right)=\frac{{OD}_{t0}{-OD}_{ti}}{{OD}_{t0}}\times 100\%$$

$$\text{Inhibition }\left(\text{\%}\right)=\frac{{{C}_{{A}_{max}}-T}_{{A}_{max}}}{{C}_{{A}_{max}}}\times 100\% $$

where OD_t0_ and OD_ti_ represent the initial and different time points of OD 405 values. C_Amax_ and T_Amax_ represent the maximum aggregation capacity of the control and experimental groups, respectively.

### Platelet activation assay

Platelets were prepared as described above. To assess the effect of QSYQ on platelet function, we pretreated platelets with QSYQ for 30 min and then added 0.5 U/mL thrombin to induce platelet activation. After 30 min, platelet samples were mixed with FITC anti-mouse/rat CD61 Antibody (1:200) and PE anti-mouse/rat CD62P Antibody (1:200) and incubated for 20 min at room temperature. Unbound dye was removed by centrifugation at 700×*g* for 5 min. The pellet was resuspended in equal volumes of buffer A. All samples were analyzed using an Attune® NxT Acoustic Focusing cytometer (Invitrogen, Thermo Fisher Scientific, USA).

To evaluate the platelet activation under immunothrombotic conditions, a whole blood-endothelial cell co-culture model was used to simulate the interaction between platelets and vascular endothelial cells [23]. 96-well plates were pretreated with 0.1% gelatin solution for 5 min, and 10,000 cells/mL were inoculated into 48-well plates. When the cells reached 70–80% confluency, whole blood pretreated with QSYQ for 30 min was added (QSYQ: 0.4 mg/mL). The co-culture was activated with LPS (1 μg/mL) for 4 h, platelet activation (PF4 and CD62P) was determined by Enzyme-linked immunosorbent assay (ELISA, Colorful-Gene Biotech, Wuhan, China) and flow cytometry.

### Bleeding time measurement

ICR mice (20–22 g) of both sexes were divided into four groups with six mice per group. Each group received one of the following treatments: saline (negative control), QSYQ (195 mg/kg), QSJG (71 mg/kg), or ticlopidine (positive control, 1 mg/kg). The drugs were administered via intragastric gavage once daily for one week. The mice were placed in a mouse holder, and their tail was immersed in 37 ℃ normal saline for 5 min. The tail was cut about 5 mm from the tip with a sharp pair of scissors, the clotting time was monitored with a stopwatch. The bleeding site was blotted with a small piece of filter paper every 15 s until the filter paper no longer showed a bloodstain [24].

### Carotid artery thrombosis model

After mice were anesthetized with isoflurane, the right carotid artery was carefully exposed and kept moist with saline. To induce thrombus formation, a Whatman paper (0.4 × 2.0 mm) presoaked in 5.5% FeCl_3_ solution was applied to cover the artery for 2 min. The carotid artery thrombi were assessed by real-time monitoring with a laser-speckle blood flow imaging system (MoorFLPI-2 Pro, Moor Instruments, United Kingdom). Each mouse was recorded for 12 min, and the occlusion time was defined as the blood flow value decreasing to 60% of normal blood flow [25]. To investigate whether SAA and SAB could inhibit thrombosis, mice were divided into the following four groups: Model (saline), SAA (20 mg/kg), SAB (20 mg/kg), and positive control (Aspirin) groups. The drugs were pre-administered for 7 days before inducing carotid artery thrombosis.

### Statistical analysis

The results were expressed as mean values and SD. We calculated significant differences using SPSS software, and we performed statistical analysis using One-way ANOVA followed by Dunnett's test. The *p* < 0.05 was taken as statistically significant between the data sets.

## Results

### QSYQ inhibited platelet aggregation triggered by three different GPCR agonists

**Fig. 1:**
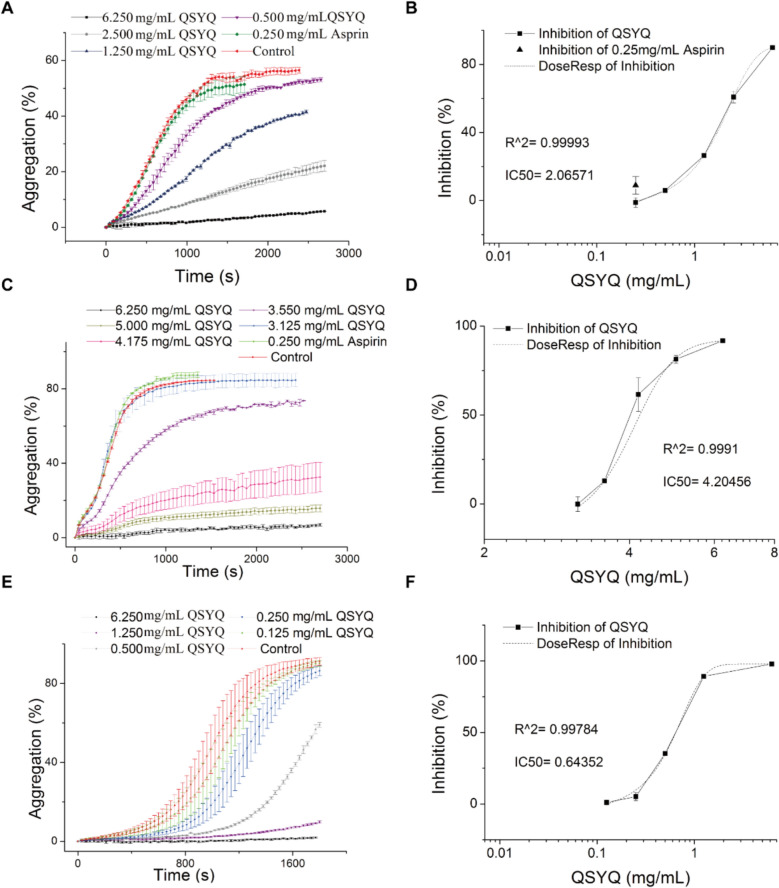
Effect of QSYQ on platelet aggregation triggered by different GPCR agonists. **A** Dose response to ADP-triggered platelet aggregation. **B** IC50 calculation of QSYQ on ADP-triggered platelet aggregation. **C** Dose response to thrombin-triggered platelet aggregation. **D** IC50 calculation of QSYQ on thrombin-triggered platelet aggregation. **E** Dose response to U46619-triggered platelet aggregation. **F** IC50 calculation of QSYQ on U46619-triggered platelet aggregation (n = 6)

To elucidate the clinical efficacy demonstrated by QSYQ in the large-scale clinical trial [18], we modified and optimized a microplate-based platelet aggregation assay for higher-throughput assessment of the anti-platelet activities. We initially assessed the potential of QSYQ to modulate aggregation triggered by the three different GPCR agonists: ADP, Thrombin, and U46619 (an analog of the TXA_2_ receptor), and the results suggested that platelet aggregation occurred in a dose-dependent manner (Figure S1B, C, and D). QSYQ inhibited platelet aggregation triggered by these three GPCR agonists in a dose-dependent manner (Fig. 1A, C, E). The IC_50_ for the agonists was 2.065 mg/mL for ADP, 4.205 mg/mL for thrombin, and 0.643 mg/mL for TXA_2_, respectively (Fig. 1B, D, F). The inhibitory effects of QSYQ at a clinically equivalent dose were greater than those of aspirin (0.250 mg/mL), a positive control in the assay.

### QSYQ prevented GPCR-agonist-triggered β-arrestin internalization

**Fig. 2:**
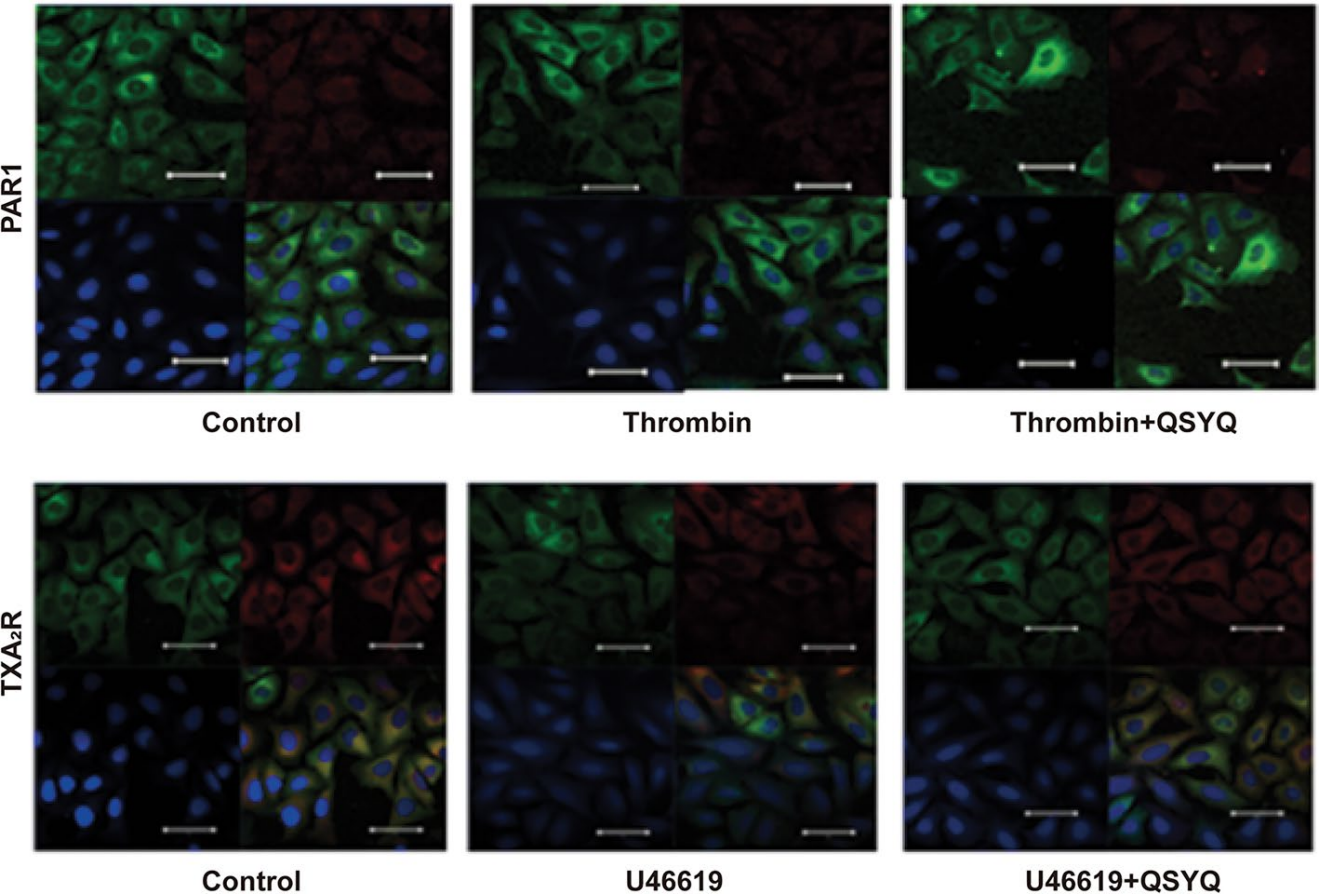
The effect of QSYQ on thrombin or U46619-mediated β-arrestin internalization was determined by immunofluorescent staining of agonist-treated cells. Color label: Nucleus (DAPI): *blue*, β-arrestin2-GFP: *green*, anti-receptor antibody: *red*. *Left panel*: no agonist control; *middle panel*: agonist (*top*: Thrombin or *bottom*: U46619) treated platelets; *right panel*: QSYQ and agonist (*top*: Thrombin or *bottom*: U46619) treated platelets. Scale bar = 100 μm. n = 3

Receptor-specific activation was determined by β-arrestin internalization assay in U2OS β-arrestin2-RrGFP cells. Immunofluorescent staining showed that the reporter cells express both thrombin and TXA_2_ receptors. Upon treatment with thrombin or U46619, a reduction in cell surface staining of the receptors was observed, alongside the formation of “pits” within green fluorescent-stained β-arrestin2. QSYQ pretreatment for 60 min significantly reversed the “pits” formation (Fig. 2).

### QSYQ reversed ADP-triggered reduction of cAMP production

**Fig. 3:**
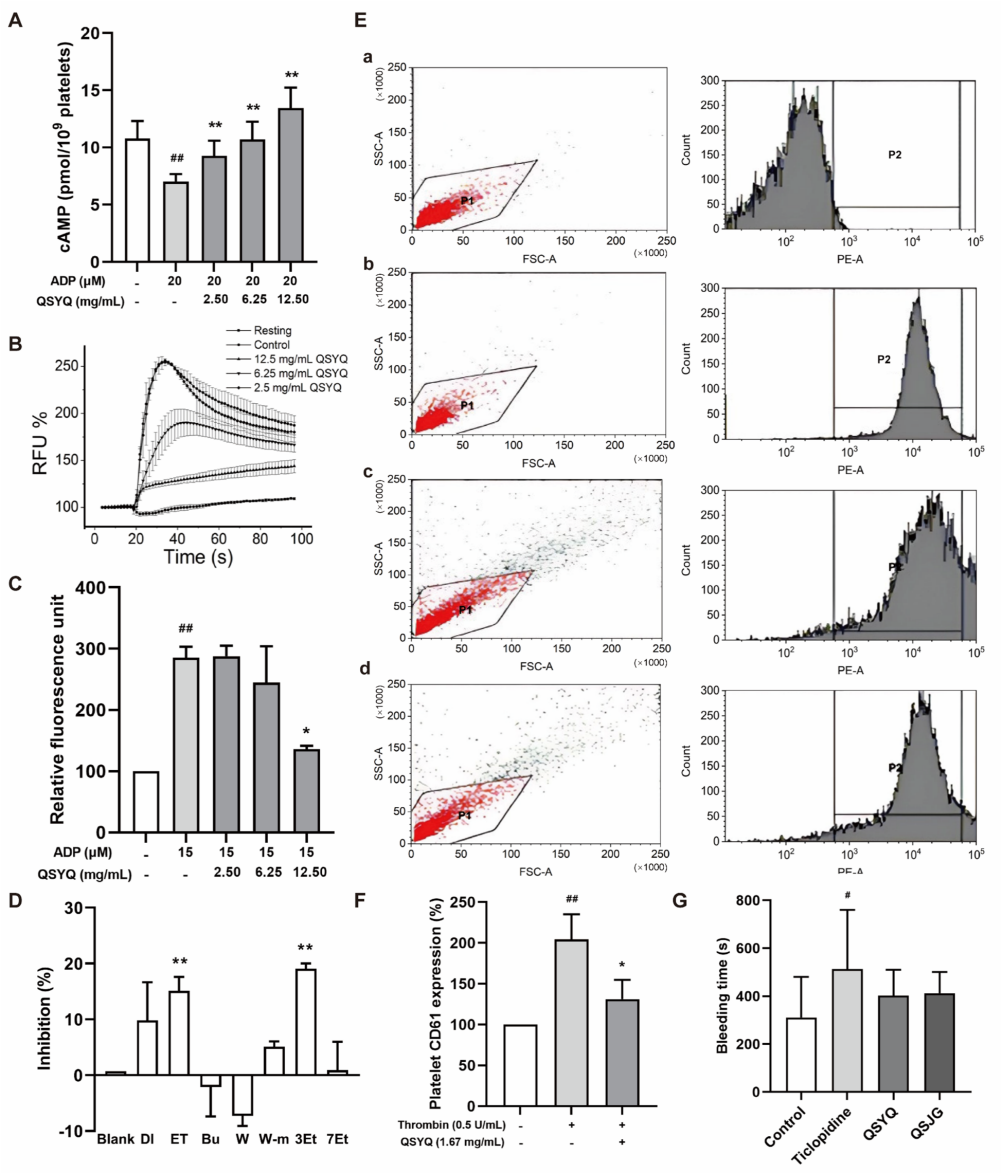
Effect of QSYQ on ADP-triggered cAMP production, Ca^2+^ influx, and bleeding time in rats. **A** Three different concentrations (2.50, 6.25, and 12.50 mg/mL) of QSYQ reversed ADP-mediated cAMP production in a dose-dependent manner (^##^*p* < 0.01: only ADP-triggered platelets activation compared to resting platelets, **p < 0.01: platelets treated with different concentrations of QSYQ compared to only ADP-triggered platelets, n = 3). **B** Kinetics of Ca^2+^ influx in rat platelet samples (n = 3). **C** QSYQ dose dependently inhibited ADP-triggered Ca^2+^ influx in isolated platelets (^##^*p* < 0.05: ADP-triggered platelets compared to resting platelets, *p < 0.05: platelets treated with different concentrations of QSYQ compared to only ADP-triggered platelets, n = 3). **D** The inhibition of Ca^2+^ influx by different QSYQ extracts in 1321N1 cells expressing P2Y_12_ receptor (*DI*: dichloromethane extract, *ET:* ethyl acetate extract, *BU*: butanol extract, *W*: water-soluble extract, *W-m*: water layer of macro-porous resin, *3Et*: 30% ethanol layer of macro-porous resin, *7Et*: 70% ethanol layer of macro-porous resin. *p < 0.05, **p < 0.01, n = 3). **E** Effect of QSYQ on platelet CD61 expression and sequential identification and isolation of its active anti-platelet components. **F** Summative data of platelet CD61 expression mean fluorescence intensity. (^##^*p* < 0.01, *p < 0.05, n = 3). **G** Effect of QSYQ on bleeding time in rat (^#^*p* < 0.05: 1 mg/kg Ticlopidine group compared to control group, n = 6)

Since cAMP production inhibition and cyclic nucleotide-dependent protein kinase activities are dependent on G_i_ or G_s_ platelet activation pathways, we investigated whether QSYQ could modulate platelet cAMP concentration. Surprisingly, 20 µM ADP reduced cAMP levels from 10.78 to 7.02 pmol/10^9^ platelets. Treatment with QSYQ at concentrations of 2.50, 6.25, and 12.50 mg/mL significantly reversed the ADP-triggered cAMP reduction in platelets in a dose-dependent manner to 2.26, 3.70, and 6.44 pmol/10^9^ platelets, respectively (Fig. 3A).

### QSYQ reversed ADP-triggered [Ca^2+^]_i_ increase

Since an increase in [Ca^2+^]_i_ accompanies the G_q_-mediated platelet activation pathway, we examined the effect of QSYQ on [Ca^2+^]_i_ levels triggered by ADP in platelets. Our observation revealed that ADP caused an immediate increase in [Ca^2+^]_i_ to 284.83%, and in ADP-triggered platelets, pretreatment with QSYQ at concentrations of 2.50, 6.25, and 12.50 mg/mL resulted in dose-dependent reductions in the relative fluorescence unit of (RFU) of [Ca^2+^]_i_ to 287.67%, 244.76%, 136.19%, respectively (Fig. 3B, C). In an independent experiment utilizing a human astrocytoma cell line (1321N1 cells) stably expressing the P2Y_12_ receptor, Ethyl acetate extract and the 30% ethanol layer from the macro-porous resin of QSYQ partially inhibited Ca^2+^ influx, to a similar extent as AR-C 66096, a potent and selective P2Y_12_ receptor antagonist (Fig. 3D).

### QSYQ inhibited platelet activation

**Fig. 4:**
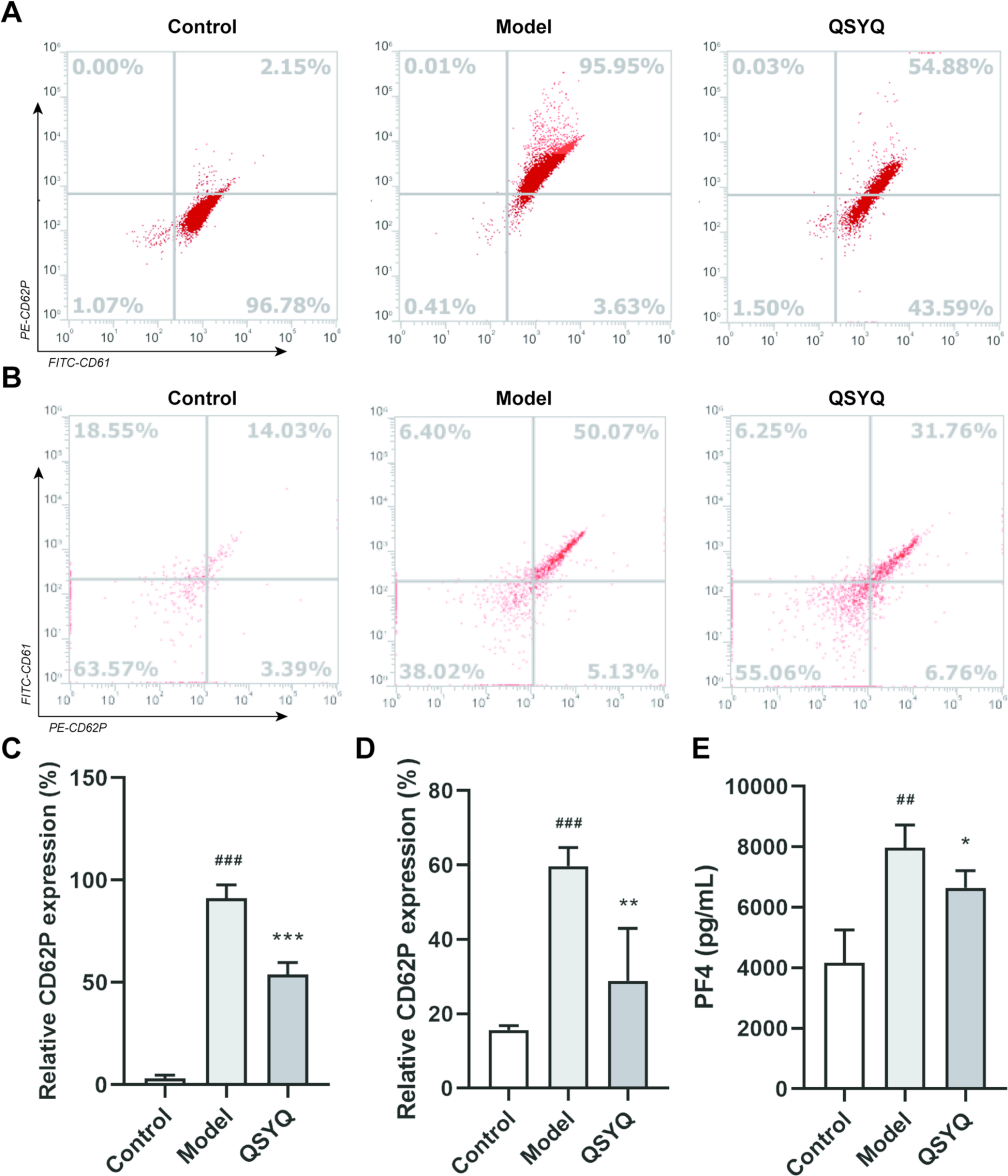
The effects of QSYQ on platelet activation. **A** Representative flow cytometry images of CD62P expression on the platelet surface. **B** Representative flow cytometry images of CD62P expression in the whole blood-endothelial cell co-culture model. **C** The quantitative expression of CD62P on the platelet surface. **D** The quantitative expression of CD62P in the whole blood-endothelial cell co-culture model. **E** The quantitative expression of PF4 by ELISA in the whole blood-endothelial cell co-culture model. Data are presented as mean ± SD. (n = 3–5), ^##^*p* < 0.01, ^###^*p* < 0.001 vs. Control. *p < 0.05, **p < 0.01, ***p < 0.001 vs. Model

To assess the functional consequences of QSYQ on platelet function, we performed flow cytometry using CD61 (Integrin β3) and CD62P antibodies. Activation of platelets by 0.5 U/mL thrombin increased the surface expression of CD61. Platelets were selected based on size and content using forward scatter (FSC) and side scatter (SSC). Histogram plots represented the surface antigen (CD61) expression in platelets. The total number of platelets determined the area content of P1. Histograms were used to quantify platelet aggregates based on the percentage of CD61-PE mean fluorescent intensity from P1 cells. Gates P2 were adjusted to include 0.5% of the isotype-control stained (Count-PE) population (Fig. 3E-a). Inactivated samples exhibited 87.5% CD61-PE expression (Fig. 3E-b). Activation with 0.5 U/mL thrombin resulted in 60% CD61-PE expression (Fig. 3E-c), and pre-incubation with QSYQ resulted in 85% CD61-PE expression (Fig. 3E-d). However, the CD61 surface expression was significantly decreased in the presence of 1.67 mg/mL QSYQ (Fig. 3E, F). Activation of platelets by 0.5 U/mL thrombin increased the surface expression of CD62P. Compared with the control group, CD62P in the model group was increased significantly (91.18%). Pretreatment with QSYQ and SAA reduced the expression of CD62P (53.86 and 71.92%), suggesting that QSYQ and SAA have anti-platelet activation effects (Fig. 4A, C).

Moreover, platelet activation is a central part of the immune thrombosis process, which connects vascular injury, coagulation, and inflammatory processes that together promote thrombosis and development. CD62P recruits and activates leukocytes and mediates platelet-leukocyte interactions, thereby enhancing thrombus stability. Studies have shown that the whole blood-endothelial cell co-culture model can simulate the interaction between blood and vascular endothelial cells, which is closer to the physiological and pathological conditions of thrombosis in vivo [23]. Compared to the model group, QSYQ significantly reduced platelet activation (CD62P and PF4) in the whole blood-endothelial cell co-culture model (Fig. 4B, D, E).

### QSYQ did not increase bleeding risk in vivo

A major challenge of anti-platelet drug development is the side effects of increased bleeding risk. To evaluate the impact of QSYQ on platelet aggregation in vivo, we compared the bleeding time of QSYQ with a known antiplatelet agent-Ticlopidine. Ticlopidine increased the bleeding time from 310.83 to 513.80 s (*p* < 0.05, n = 6), QSYQ and QSJG did not show a significant increase in bleeding time (402.00 and 421.00 s, *p* = 0.336 and *p* = 0.269, respectively (n = 6, Fig. 3G).

### The chromatographic confirmation of SAA in QSYQ and its other pharmacological constituents

**Fig. 5:**
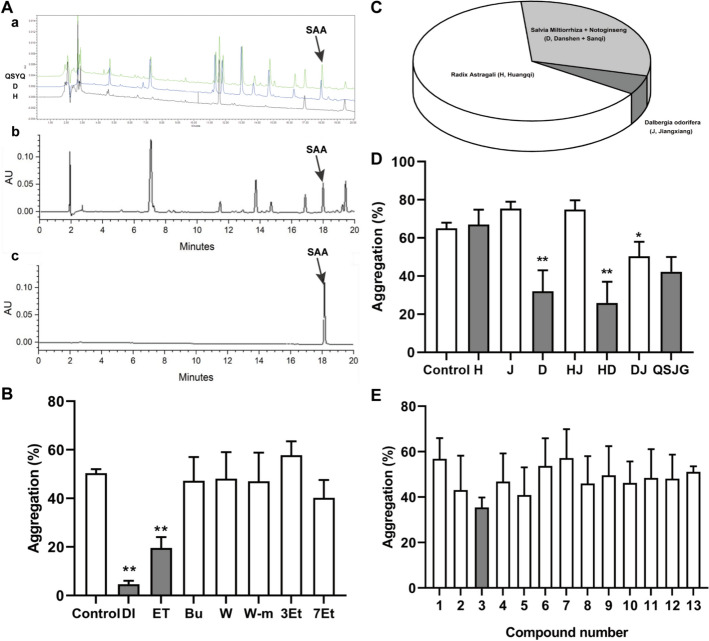
Platelet-aggregation activity-based screen of QSYQ fractions and compound library. **A** A diagram of QSYQ separation. **a** UPLC profile of QSYQ full extract (*green*), Radix Astragali (H, Huangqi) extract (*blue*), and *Salvia Miltiorrhiza* + *Notoginseng* (D, Dansen + Sanqi) extract (*black*). **b** UPLC profile of the Ethyl acetate fraction of QSYQ. **c** UPLC profile of the SAA standard. The arrow indicates the position of SAA. **B** Relative anti-platelet activities of the chemical fractionations of QSYQ full extract (**p < 0.01, compared to control, n = 3). **C** The pie chart of QSYQ is composed. **D** Relative anti-platelet activities of composite QSYQ extracts, (*Radix Astragali* (H, Huangqi), *Salvia Miltiorrhiza* and Notoginseng (D, Danshen + Sanqi), Dalbergia odorifera (J, Jiangxiang), QSYQ full extracts (QSJG), **p < 0.01, *p < 0.05 compared to QS, n = 3). **E** Relative anti-platelet activities of single chemical compounds. 1. Tanshinone I. 2. Tanshinone IIA. 3. Salvianolic acid A. 4. Salvianolic acid B. 5. Salvianolic acid C. 6. Protocatechuic aldehyde. 7. Protocatechuic acid. 8. Rosmarinic acid. 9. Dihydrotanshinone I. 10. Lithospermic acid. 11. Danshensu. 12. Caffeic acid (see Table 1, n = 3)

To detect and quantify SAA in QSYQ and its constituents, we performed UPLC analysis on QSJG (Green), *Radix Astragali* (H, Huangqi) extract (Blue), and *Salvia Miltiorrhiza* + *Notoginseng* (D, Danshen + Sanqi) extract (Black). SAA was identified by the retention time of the standard sample (Fig. 5A-a, A-c). SAA was present only in Danshen + Sanqi and was absent from Huangqi. The ethyl acetate fraction also demonstrated the presence of SAA, as confirmed by UPLC analysis (Fig. 5A-b).

### Identification of SAA as an active antiplatelet component by a sequential screen of QSYQ

To identify the anti-platelet active component of QSYQ, we employed microplate-based screening of the components, extracts, and compounds of QSYQ. QSYQ extracts were chemically fractionated using polar or nonpolar solvents, followed by a platelet aggregation assay. Among the seven fractions, Dichloromethane (DI) and Ethyl acetate (ET) fractions exhibited the highest inhibitory activity in platelet inhibition (95.40 and 80.45%, respectively, Fig. 5B). As shown in the pie chart, QSYQ consists of *Radix Astragali* (H, Huangqi), *Salvia Miltiorrhiza* + *Notoginseng* (D, Danshen + Sanqi), and *Dalbergia odorifera* (J, Jiangxiang) (Fig. 5C). These components were screened individually or in combination for their effects on platelet aggregation. Compared to the control group, which caused 65.16% platelet aggregation, neither **H** (67.05%), **J** (75.42%), nor the **HJ** combination (74.95%) showed a significant difference. However, **D** (32.05%), **HD** (25.93%), and **DJ** (50.35%) showed significant antiplatelet activity (**p < 0.01, *p < 0.05, n = 3), which was comparable to the **QSJG** (42.29%). The most active antiplatelet activity of QSYQ was likely attributed to *Salvia miltiorrhiza* (Fig. 5D). Additionally, certain compounds from *Salvia miltiorrhiza* were further screened. Among the 12 major compounds screened (see Table 1 for details), only SAA showed stronger inhibition (30.71%, Fig. 5E), confirming it as the main antiplatelet component in QSYQ.

**Table 1:** Inhibition of platelet aggregation by 12 QSYQ-derived compounds

Compound number	Chemical name	Concentration (μM)	Platelet aggregation
1	Tanshinone I	10	56.83 ± 9.11
2	Tanshinone II A	10	43.07 ± 15.15
3	Salvianolic acid A	10	35.43 ± 4.42
4	Salvianolic acid B	10	46.83 ± 12.40
5	Salvianolic acid C	10	40.87 ± 12.20
6	Protocatechuic aldehyde	10	53.71 ± 12.16
7	Protocatechuic acid	10	57.19 ± 12.68
8	Rosmarinic acid	10	45.98 ± 12.03
9	Dihydrotanshinone I	10	49.54 ± 12.91
10	Lithospermic acid	10	46.26 ± 9.47
11	Danshensu	10	48.45 ± 12.65
12	Caffeic acid	10	48.10 ± 10.63
13	–	–	51.13 ± 2.42

### SAA did not reverse ADP-triggered cAMP production

**Fig. 6:**
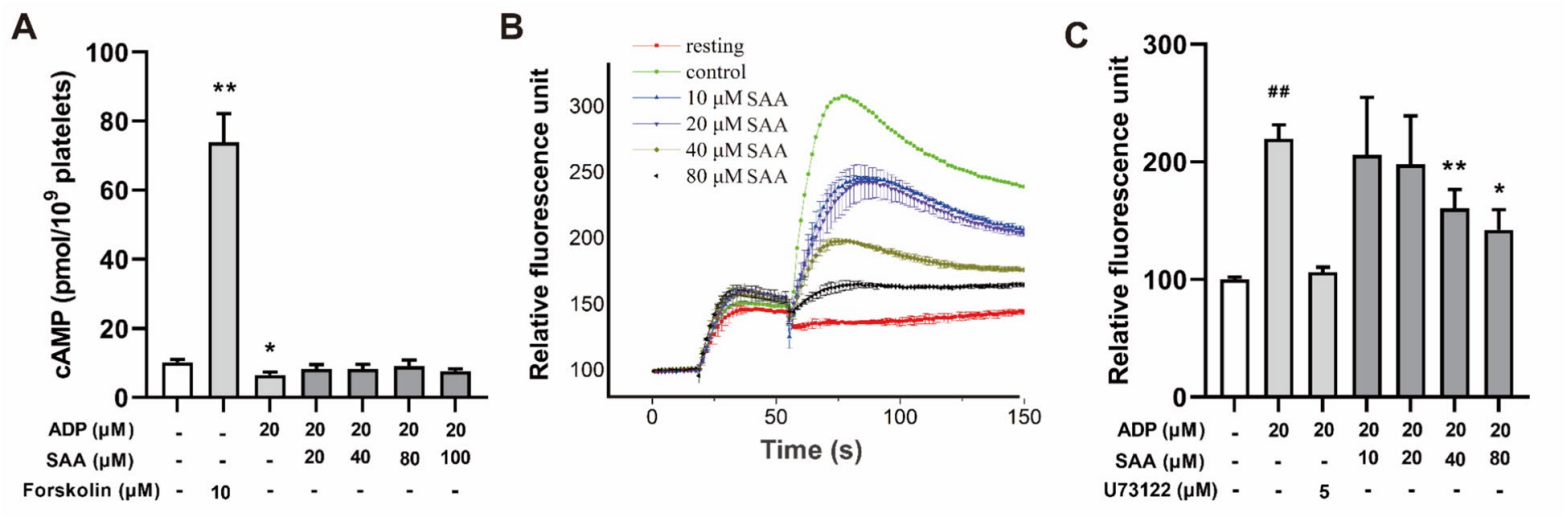
Effect of SAA on ADP-triggered cAMP production and Ca^2+^ influx. **A** The bar graph shows the production of cAMP in each group. Forskolin caused a significant cAMP increase, and 20 μM ADP reduced cAMP production (*p < 0.05, **p < 0.01, compared to the resting platelets, n = 4). **B** Kinetics of Ca^2+^ influx in isolated rat platelets. **C** Effect of SAA on ADP-triggered Ca^2+^ influx in isolated platelets. Four different concentrates (10, 20, 40, and 80 μM) of SAA reversed ADP-triggered [Ca^2+^]i influx in a dose dependent manner (^##^*p* < 0.05: ADP-triggered platelets compared to resting platelets, **P < 0.01 *p < 0.05: platelets treated with different concentrations of SAA compared to only ADP-triggered platelets, n = 4). **D** Representative images and quantitation of positive CD62P in platelets (n = 4–6). Data are presented as mean ± SD. ^##^*p* < 0.01, ^###^*p* < 0.001 vs. Control. *p < 0.05, **p < 0.01, ***p < 0.001 vs. Model

The cAMP production was determined to assess the impact of QSYQ or SAA on downstream events of ADP-triggered platelet activation. A positive control, Forskolin, caused a substantial increase in cAMP levels (4.47-fold), whereas 20 μM ADP significantly reduced the cAMP production (*p < 0.05). SAA at 20–100 μM did not reverse cAMP production triggered by the ADP (Fig. 6A).

### SAA reversed ADP-triggered [Ca^2+^]_i_ influx

The capacity of SAA to reduce the [Ca^2+^]_i_ influx induced by ADP (20 μM) was also investigated. While the positive control drug, a phospholipase C (PLC) inhibitor U73122 (5 μM) inhibited [Ca^2+^]_i_ influx by 52.56%, SAA at concentrations of 10, 20, 40, and 80 μM inhibited the [Ca^2+^]_i_ influx by 8.39, 12.37, 28.53, 37.16%, respectively (Fig. 6B, C).

### Confirmation that SAA is a natural ADP receptor antagonist, binding to P2Y_1_ with higher affinity than to P2Y_12_

**Fig. 7:**
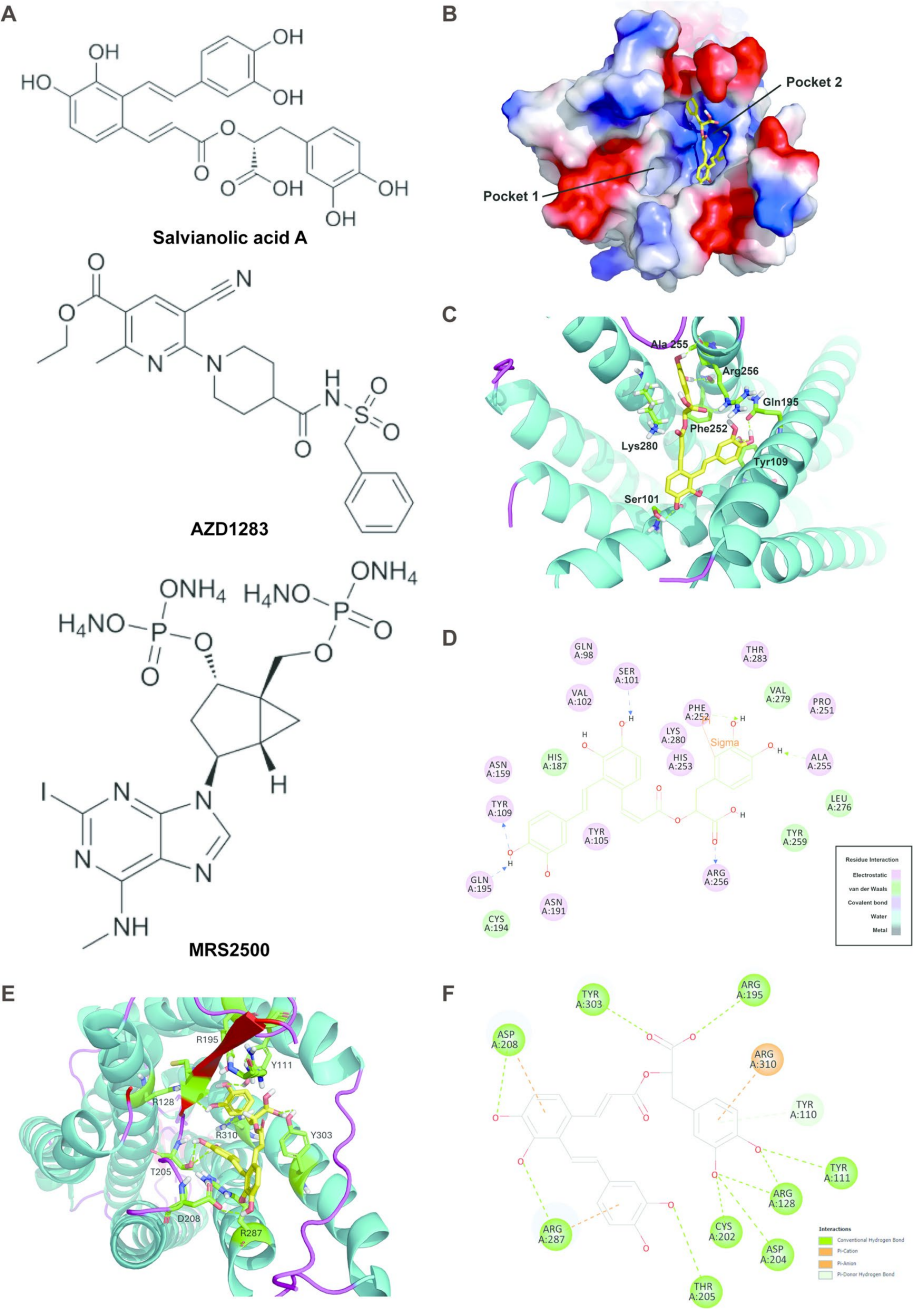
Molecular docking of SAA with P2Y_1_ and P2Y_12_ receptors. **A** Molecular structures of SAA, P2Y_12_ antagonist AZD1283 and P2Y_1_ antagonist MRS2500. **B** Docking pose of SAA in the binding pocket of P2Y_12_ receptors. **C** Binding mode of SAA with the key amino acid residues of P2Y_12_ receptors. **D** Interactions between SAA and the residues in the binding pocket of P2Y_12_ receptors. **E** Binding mode of SAA with the key amino acid residues of P2Y_1_ receptors. **F** Interactions between SAA and the residues in the binding pocket of P2Y_1_ receptors

Molecular docking was performed to determine further whether SAA reverses ADP-triggered platelet aggregation through the ADP receptor pathway. SAA and AZD1283 were docked into the P2Y_12_ protein, where AZD1283, as a co-crystallized ligand of the P2Y_12_ receptor (PDB:4NTJ), served as a validating molecule and positive control. SAA and MRS2500 were docked into the P2Y_1_ protein, where MRS2500, as a co-crystallized ligand of the P2Y_1_ receptor (PDB: 4XNW), served as a validating molecule and positive control (Fig. 7A) [26, 27]. AZD1283 was successfully redocked into P2Y_12_ with an RMSD of 0.8111 which was compared with the co-crystallized structure, this docking yielded a total score of 7.8105. As shown in Fig. 7B, SAA was also docked into the binding pocket of P2Y_12_ in a similar position with a total score of 10.0289. Between the SAA and P2Y_12_ receptor, there were 6 Hydrogen bonds formed with the following amino acid residues: Ser101, Tyr109, Gln195, Phe252, Ala255, and Arg256 (Fig. 7C, D). Where the C28 in the aromatic ring of SAA interacts with the phenyl group of Phe252 through a Sigma-Pi interaction. Additionally, SAA interacts with amino acid residues His187, Tyr259, Leu276, and Val279, resulting in van der Waals forces between them. SAA was then docked into the binding pocket of P2Y_1_ with a total score of 10.4179. Between the SAA and P2Y_1_ receptor, there were 10 Hydrogen bonds formed with 9 amino acid residues: Tyr111, Arg128, Arg195, Cys202, Asp204, Thr205, Asp208, Arg287, Tyr303 (Fig. 7E, F). Compared with the P2Y_12_ receptor, SAA forms more hydrogen bonds with the P2Y1 receptor, which suggests SAA has a higher binding affinity with P2Y_1_ than P2Y_12_ receptor.

### SAA inhibited ADP-triggered platelet aggression

**Fig. 8:**
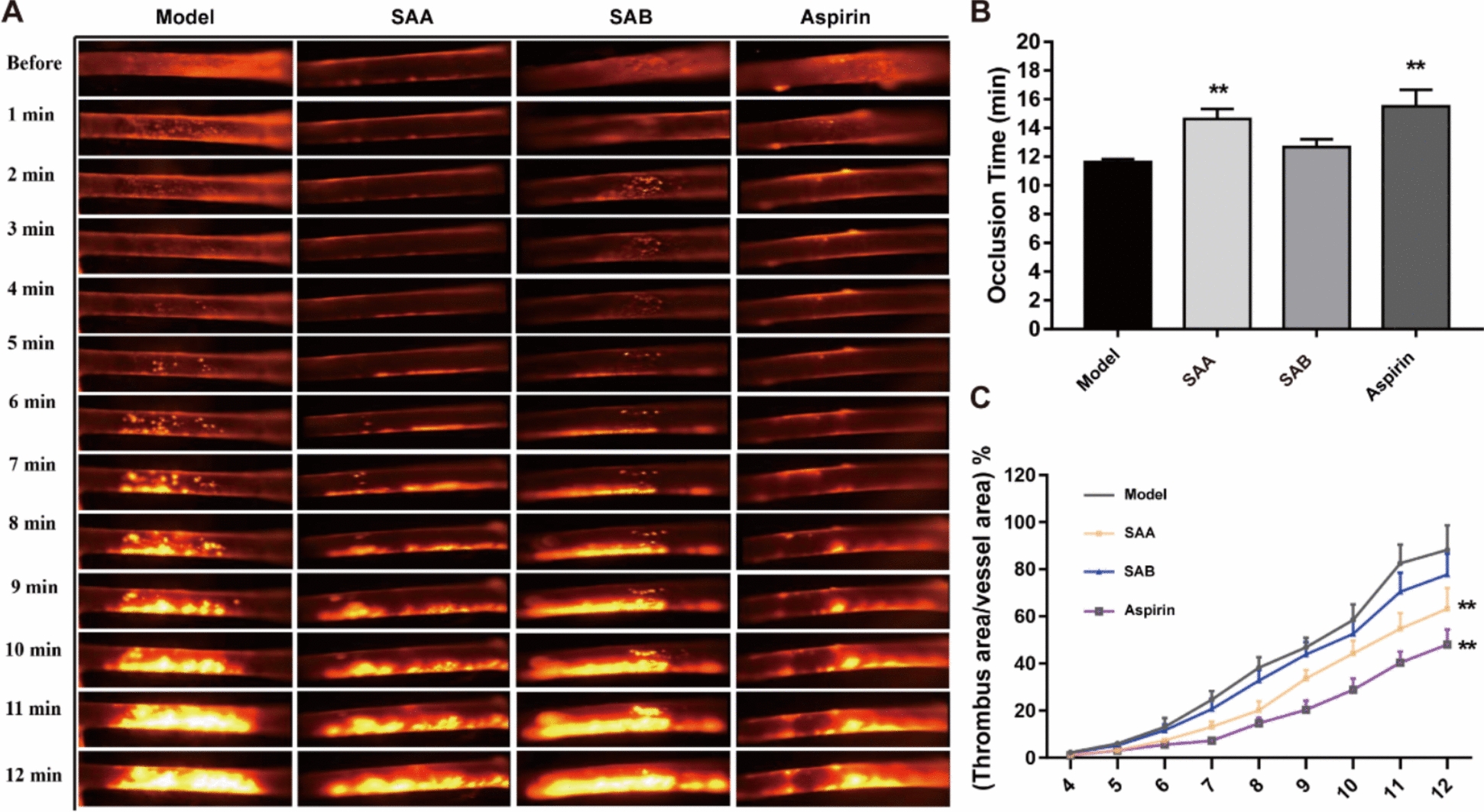
SAA improved carotid arterial thrombosis in mice. **A** Representative images of speckle Doppler flow imaging and time plot of average blood flow per minute in different treatment groups. **B**, **C** Effects of SAA and SAB on occlusion time and the rate of thrombus area/vessel area. Data are presented as mean ± SD. **p < 0.01, compared to Model (n = 6–8)

In a ferric chloride (FeCl_3_)-induced thrombosis model [28, 29], following a 1-min treatment with FeCl_3_, a 30-min in vivo detection of thrombus formation was conducted, and pictures were taken at each minute during this period (from 0 to 12 min). Vessel occlusion time and thrombus area were recorded to evaluate the antithrombotic effects of the two major QSYQ ingredients, SAA and SAB (Fig. 8A). The average occlusion time (complete occlusion) in the model group was nearly 12 min. SAA and aspirin significantly reduced thrombus formation and vessel occlusive time, as thrombus area/vessel area (%) was markedly reduced after pretreatment with SAA and aspirin (Fig. 8A, B). In comparison, SAB exhibited only a modest anti-thrombotic effect (Fig. 8C).

## Discussion

Traditional Chinese medicines have promising potential and advantages for thrombotic diseases. QSYQ, a widely used cardiovascular medicine in China, has demonstrated strong efficacy in the prevention and treatment of myocardial infarction and heart failure [17–19]. Our recent study demonstrated that QSYQ had good antiplatelet activity in an ischemic stroke setting. In carotid arterial thrombosis mice, QSYQ and its YQ/HX components inhibited thrombus formation, prolonged vessel occlusion time, reduced circulating leukocytes, and P-selectin expression. PLA formation and platelet/leukocyte adhesion to the endothelial cell were also inhibited by QSYQ and its YQ/HX components in vitro [30]. Various compounds in QSYQ have also shown favorable antiplatelet effects. SAA is a water-soluble component from the root of *Salvia miltiorrhiza*, which prevents thrombosis with a mild inhibitory effect on platelet aggregation. Studies have shown that SAA (0.1 mg/mL) reduced platelet activation and that SAA significantly inhibited P-selectin expression and fibrinogen binding induced by ADP and thrombin stimulation. It also inhibited the formation of platelet-leukocyte aggregates [31, 32]. Rosmarinic acid exhibited potent inhibitory effects on the platelet aggregation induced by arachidonic acid (AA) induced platelet aggregation and the enzymatic activity of ERp57 [33]. SAB and lithospermic acid markedly decrease the chance of thrombosis by regulating the NF-κB/JNK/p38 MAPK signaling pathway in response to TNF-α [34]. Notoginseng has been reported to regulate platelet function and coagulation factor activity. It also promoted rapid clotting at sites of tissue damage while facilitating the timely dissolution of fibrin, preventing thrombosis, and effectively controlling bleeding [35, 36].

In the present study, we investigated QSYQ’s effects on platelet GPCRs. QSYQ dose-dependently inhibited platelet aggregation induced by various GPCR agonists. It significantly reversed ADP-triggered reduction in cAMP production and increased [Ca^2+^]_i_ influx, prevented GPCR agonist-triggered β-arrestin internalization, and inhibited surface expression of CD61 and CD62P. We took a reverse pharmacology approach to systematically identify QSYQ components based on their GPCR-dependent antiplatelet activities. The extracts of *Salvia miltiorrhiza* and *Notoginseng* significantly inhibited platelet aggregation. The UPLC analysis revealed the presence of SAA in the extracts of *Salvia miltiorrhiza* and *Notoginseng*, and SAA had significant anti-platelet activity. This led to the identification of SAA as one of the major active components for platelet inhibition in QSYQ. Platelet aggregation assays showed that the dichloromethane fraction of QSYQ was the most effective in inhibiting platelet aggregation. Whereas SAA is a water-soluble component and is mainly distributed in aqueous extracts, the dichloromethane extract had very low content of SAA, but it had the strongest antiplatelet aggregation ability, which may be due to the highest content of QSYQ antiplatelet aggregation active components in the dichloromethane extract. However, which component played a role needs further verification.

It was shown that QSYQ modulates the cAMP-CREB-BDNF pathway, significantly inhibits the upregulation of PDE4 (cAMP-degrading enzyme), and increases CREB phosphorylation, and ameliorates cognitive deficits associated with heart failure. Molecular docking showed that the core components of QSYQ (Dihydrokaranone, Ginsenoside Rh2) bound well to cAMP pathway targets (PDE4, CREB), suggesting that it may be these components play a role in modulating cAMP [37]. Moreover, although SAA has documented cardiovascular protective roles [38, 39], and prior studies linked its antiplatelet effects to elevated phosphoinositide 3-kinase (PI_3_K) inhibition [32, 40]. Nonetheless, our results indicated that SAA did not reverse the reduction in ADP-triggered cAMP production. This inconsistency potentially stems from the different concentrations of SAA utilized in the present and earlier studies. In our cAMP assay, we used SAA at concentrations ranging from 10 to 80 μM, whereas the earlier study employed higher concentrations of 200–1000 µg/mL, equivalent to 400–2000 μM [40]. We suggested that our experimental conditions more closely mimic a physiological environment. In the present study, our results suggested that SAA selectively regulated the second messenger downstream of ADP-induced platelet activation, and dose-dependently inhibited ADP-induced increases in [Ca^2+^]_i_ without modulating cAMP levels.

The antithrombotic effects of SAA, SAB, and SAC, both in vitro and in vivo, have been previously reported by us and others [40–42]. Importantly, Liu et al. showed the binding poses of SAA within the binding sites of P2Y_1_ and P2Y_12_ receptors. For the P2Y_1_ receptor, the binding pose of SAA was like that of the co-crystallized antagonist MRS2500, consistent with their function as P2Y_1_ antagonists. At the P2Y_12_ receptor, the binding pose of SAA mimicked that of the co-crystallized antagonist AZD1283, contrasting with the co-crystallized agonist 2MeSADP. Furthermore, direct binding assay showed that Ki of SAA for P2Y_12_ is 20.3 ± 1.5, whereas for P2Y_1_ is 14.7 ± 2.4. These results were consistent with the evidence that SAA acts as a selective antagonist of the P2Y_1_ receptor [43]. Our molecular docking assay confirmed the possibility of SAA modulating Ca^2+^ concentration by inhibiting the P2Y_1_ receptor. SAA exhibited high binding affinity to P2Y_1_, indicating successful docking into the binding pocket, and SAA was an inhibitor of P2Y_1_ receptors, thereby inhibiting platelet aggregation through reducing [Ca^2+^]_i_ influx. To prove our hypothesis, we carried out an ADP-triggered [Ca^2+^]_i_ influx assay and found that at concentrations of 20–80 μM, SAA can significantly reduce [Ca^2+^]_i_ influx in a dose-dependent manner. Therefore, these results indicated that the antiplatelet activity of SAA was mediated by the G_s_/IP_3_ pathway, not the G_i_/AC pathway. And our study revealed that multiple components in QSYQ can act through both G_i_/cAMP and G_q_/Ca^2+^ pathways. Furthermore, we identified SAA as one of the active components exerting its effects through the G_q_/IP_3_ pathway. This provided a mechanistic clarification for the complexity of QSYQ.

Mitogen-activated protein kinases (MAPKs) are signaling molecules downstream of Gq/IP_3_, transmitting signals activated by Gq proteins to the nucleus, thereby regulating a variety of cellular functions. Specifically, Gq proteins produce IP_3_ and DAG through the activation of phospholipase C (PLC), where IP_3_ binds endoplasmic reticulum IP₃ receptors (IP₃R) and triggers Ca^2^⁺ release into the cytoplasm, while DAG activates protein kinase C, processes that further activate MAPK signaling pathways [44]. MAPKs can affect arachidonic acid (AA) release by regulating phospholipase A2 (cPLA2) activity, which in turn promotes TXA_2_ synthesis [45]. It has been shown that activation of ERK1/2 and p38 MAPKs was essential for TXA_2_ production, especially in the GPIb-mediated pathway [46]. In addition, MAPKs further affect the activation of cPLA2 by regulating the phosphorylation status of C3G, thereby amplifying TXA_2_ synthesis [47]. Our findings indirectly suggested that QSYQ/SAA inhibited Gq activation by antagonizing the P2Y_1_ receptor, reduced IP₃ production, decreased [Ca^2^⁺]i, and attenuated PKC activation, possibly down-regulating MAPK phosphorylation. Although MAPKs and TXA_2_ were not directly probed in the present study, it is reasonable to hypothesize that QSYQ/SAA may synergistically block the platelet self-amplification mechanism by inhibiting the activation of MAPKs downstream of Gq and decreasing TXA_2_ production.

Moreover, although studies have shown that SAB has been shown to inhibit platelets as a P2Y_12_ antagonist [43]. Our results confirmed the antithrombotic activity of SAA. However, we were unable to detect antithrombotic activity of SAB. We believe that this discrepancy is due to the source of platelets in the two studies, in which Liu's group used human platelets while we used rat platelets. ADP receptor or its downstream signaling pathways might have different sensitivity or response in humans and rats. Our previous research results have demonstrated that the interaction between these salvianolic acid compounds affected the anti-platelet activity either synergistically or antagonistically. Although SAC alone did not affect thrombin-induced platelet aggregation, a combination of SAA and SAC synergistically inhibited platelet aggregation in vitro, while SAB counteracted this effect. This apparent controversy remains to be further explored [21].

Given the multiherbal composition nature of QSYQ, interactions of its components may alter the absorption, distribution, metabolism, and excretion (ADME) processes, thus affecting the bioavailability and intensity of action of the pharmacodynamic substances. In an in vivo exposure study of QSYQ after oral administration to rats, Fan et al. showed that the major metabolites in urine, blood, and bile were flavonoids and phenolic acids. At the same time, a variety of ginsenoside compounds, such as ginsenoside Rb1, ginsenoside Rd, ginsenoside Re, ginsenoside Rg1, and Ginsenoside R1, were detected in the urine and blood. *Dalbergia odorifera* oil was only distributed in the small intestine, liver, and adipose tissue due to its high fat solubility. Flavonoids were oxidized/glucuronidated by the hepatic enzyme CYP450. Ginsenoside compounds were hydrolyzed by intestinal flora to secondary glycosides to enhance activity. Water-soluble metabolites were mainly excreted via the kidneys, saponin prototypes and metabolites were excreted from the bile [48]. Studies have shown that SAA was poorly absorbed in animals, with an average absorption rate of 20–30%. Its absorption occurred mainly in the gastrointestinal tract and was higher in the small intestine and liver. In addition, SAA can also be detected in brain tissue. SAA was metabolized in vivo mainly by methylation and glucuronidation binding. The metabolism of the oral route was consistent with that of the intravenous route, and the methylated metabolites have antiplatelet aggregation activity. SAA was excreted in bile, urine, and feces, with approximately 0.78% of the administered dose excreted in the feces, followed by bile and urine. The absolute bioavailability of SAA was low (0.39–0.52%) [49].

Overall, our current study further illustrates that QSYQ, as a multi-target GPCR antagonist (targeting thrombin receptor, TXA_2_ receptor, and the purinergic receptor family, such as P2Y_1_ and P2Y_12_), has significant importance in selectively antagonizing P2Y_1_ receptors, mainly reflected in the following aspects. (1) It is the key initial step for precise intervention in platelet activation: The P2Y_1_ receptor is a key initiating receptor for ADP-induced platelet activation. After ADP binds to P2Y_1_, PLC-β is activated by Gq protein, leading to the generation of IP_3_ and the release of intracellular calcium ions ([Ca^2^⁺]_i_) [50]. This is necessary for platelet shape changes (from disc-shaped to spherical and extending pseudopodia), initial aggregation, and weak activation (such as partial activation of GPIIb/IIIa receptors) [51]. Selective antagonism of P2Y_1_ can directly and efficiently block the core initial signal of platelet activation. This is more effective in inhibiting platelet activation at the source than simply blocking downstream or synergistic pathways (P2Y_12_). (2) It overcomes the limitations of P2Y_12_ single target inhibition: The widely used P2Y_12_ antagonists in clinical practice, such as clopidogrel, ticagrelor, and prasugrel, are the cornerstone of antiplatelet therapy [52]. However, some patients have "clopidogrel resistance" or high residual platelet reactivity, resulting in poor therapeutic efficacy [53, 54]. ADP achieves maximum activation and stable aggregation of platelets through the synergistic effect of P2Y_1_ and P2Y_12_ [55, 56]. The P2Y_12_ signal (Gi-mediated) is mainly responsible for amplifying and stabilizing aggregation, promoting dense particle release (releasing more ADP), and inhibiting adenylate cyclase (reducing cAMP levels) [57]. When only blocking P2Y_12_, the initial calcium signal and weak aggregation mediated by P2Y_1_ still exist. Selective antagonism of P2Y_1_ can eliminate this "residual activation" and form complementary or even synergistic effects with P2Y_12_ antagonism, possibly overcoming the resistance of some P2Y_12_ inhibitors and providing stronger antiplatelet effects. (3) It provides more comprehensive antithrombotic protection: Thrombosis is a complex process involving multiple agonists (ADP, TXA_2_, thrombin, collagen, etc.) and receptors [58]. The multi-target antagonist QSYQ itself provides broad-spectrum inhibition by blocking multiple receptors. Among them, selective antagonism of P2Y_1_ is a crucial part of its multi-target effect. It directly cuts off the upstream of the ADP pathway, weakening the initial response ability of platelets to ADP, making it more difficult to activate and form a stable thrombus core. This, together with the antagonistic effects of other targets such as the amplification and stabilization of P2Y_12_ and the rapid calcium influx of P2X_1_, constitutes a more comprehensive antiplatelet and antithrombotic network. (4) It has at least a theoretical advantage of potential reduction in bleeding risk: Traditional potent antiplatelet drugs, especially potent P2Y_12_ antagonists and GPIIb/IIIa antagonists, often come with a higher risk of bleeding [59]. In theory, selective inhibition of P2Y_1_ may have more favorable safety, in first, a gentler effect: P2Y_1_ mainly mediates initial and reversible aggregation and weak activation, while P2Y_12_ and GPIIb/IIIa mediate the key steps of stable and irreversible aggregation and thrombus formation. Inhibiting the initial steps may have a relatively small impact on hemostatic function [60]. Secondly, by not affecting the cAMP pathway: P2Y_1_ does not directly affect the key molecule cAMP, which maintains endothelial integrity and inhibits platelet activation, through the Gq signal (the Gi signal of P2Y_12_ inhibits cAMP elevation). Maintaining high levels of cAMP can help protect endothelial function and may reduce bleeding tendency [61]. As a multi-target drug, the overall bleeding risk of QSYQ requires a comprehensive evaluation of the effects of all targets. But the selective P2Y_1_ antagonistic effect included in it is believed to help it achieve strong antithrombotic effects while maintaining a relatively acceptable risk of bleeding (more clinical data is needed to confirm). (5) It provides key mechanisms under targeted pathological conditions: Under certain pathological conditions (such as diabetes and chronic kidney disease), the reactivity of platelets to ADP may be enhanced [62, 63]. The P2Y_1_ signaling pathway may play a more important role. Selective antagonism of P2Y_1_ may demonstrate unique therapeutic advantages in these specific populations.

## Conclusions

In conclusion, the present study showed that QSYQ, an effective natural combination antiplatelet agent, significantly inhibited platelet aggregation through multiple platelet GPCRs, affecting both cAMP production and [Ca^2+^]_i_ influx in vitro and in vivo, without increasing bleeding time. Furthermore, one of its active components, SAA, selectively acted via the P2Y_1_/G_q_/IP_3_ pathway. Our findings contributed to a deeper understanding of the multitargeting effect of QSYQ, which could aid in developing novel pharmaceutical strategies for treating thrombotic disorders.

## Supplementary Information

Additional file 1.

## Data Availability

All the data generated or analyzed during this study are included in the published article and its Supplementary Files.

## Abbreviations

QSYQ: Qishen Yiqi dripping pill
GPCRs: G protein-coupled receptors
SAA: Salvianolic acid A
SAB: Salvianolic acid B
cAMP: Cyclic adenosine monophosphate
[Ca^2+^]_i_: Intracellular Ca^2+^ concentration
1321N1 cells: Human astrocytoma cell line
QSJG: QSYQ extracts
