# Supplementary material for: Identification of salvianolic acid A as an ADP receptor-selective and Gq/IP3 pathway-mediated anti-platelet component in Qishen Yiqi
Source: Chin Med. 2025 Oct 6;20:167. doi: 10.1186/s13020-025-01188-w (PMC12498442; doi:10.1186/s13020-025-01188-w)
Supplement: Supplementary file 1 — Additional file 1. [file 13020_2025_1188_MOESM1_ESM.docx]

**Supplemental Information**

**Rapid platelet aggregation assay based on microplate**

Platelet aggregation state can be characterized by its absorbance change at 405 nm. Washed platelets were prepared and re-suspended in buffer A, diluted to create a concentration gradient, and 100 µL of platelets was added into a 96-well microplate in triplicate, and the OD405 values of platelets were measured by FlexStation^®^ 3. Platelets are easy to activate *in vitro*, but the relationship between OD405 value and platelets concentration is acceptable (**Figure S1A**). Platelet concentration of 1 × 10^8^ platelets /mL is taken as the ideal concentration for control. The percentage of platelet aggregation can be calculated according to OD405 value and OD405 value of the initial platelets is similar in each repeatable test.

**cAMP ELISA analysis**

Serum of piglets were collected and the change of d-lactate (d-LA) and diamine oxidase (DAO) concentrations between different groups were detected by Enzyme-linked immunosorbent assay (ELISA) according to the manufacturer’s protocol (Colorful-Gene Biotech, Wuhan, China). The correlation between the cAMP concentration and OD405 value was described by the equation, with an R^2^ value of 0.99993 (**Figure S2A**). In cAMP ELISA test, PGE1 at concentrations of 0.1, 1, and 10 μM increased cAMP production in a dose-dependent manner (**Figure S2B**). As a positive control, PGM1 was included in each repeated test during the cAMP measurement of QSYQ (***p* < 0.01: compared to resting platelet, *^##^p* < 0.01: 0.1 μM PGE1 compared to 1 μM PGE1, *^$$^P* < 0.01: 1 μM PGE1 compared to 10 μM PGE1).

**P2Y_12_/Gα15-mediated Ca^2+^ influx**

1321N1 cells were seeded in a 384-well black-wall, clear-bottom plate at a density of 20,000 cells per well in 20 µL of growth medium,18 hours prior to the experiment and maintained at 37°C, 5% CO_2_. For the antagonist assay, 20 µL of dye-loading solution and 10 µL of candidate drugs or control antagonist (AR-C 66096) solution were added into the well. Then the plate was placed into a 37°C incubator for 60 min, followed by 15 min at room temperature. Finally, 12.5 µL of ADP was added into the respective wells of the assay plate. ADP was added to the reading plate at 20 s and the fluorescence signal was monitored for an additional 100 s (from 21 s to 120 s).

**[Ca^2+^]_i_ measurement**

Platelets were obtained as previously described and subjected to centrifugation at 200 g for 8 min, then re-suspended to a final concentration of 1 × 10^8^ platelets/mL in modified Tyrodes buffer (150 mM NaCl, 3 mM KCl, 1 mM MgCl_2_, 10 mM HEPES, 0.5 mM dextrose, 0.1% BSA, pH 7.4), added with 10 U/mL heparin, 0.2 U/mL Apyrase, 1 μM PGI_2_, and incubated for 8 min at room temperature. Platelets were re-suspended in modified Tyrodes buffer containing 2.5 mM probenecid, 0.02% pluronic and 10 μM Fluo-3/AM, 0.5 μM PGI_2_, 0.02 U/mL Apyrase, and shaken for 30 min at 37°C, 100 rpm. Different concentrations of drugs were added to platelets and incubated for 15 min. Subsequently, 0.5 μM PGI_2_ was added and unbound dye was removed by repeating the washing step. Platelets were finally re-suspended in Tyrodes buffer and dispensed into a 96-well microplate. [Ca^2+^]_i_ was measured using Flou-3/AM fluorescence in flex mode of FlexStation^®^ 3 (Ex 488 nm, Em 525 nm), with the relative fluorescence unit (RFU) was read every 1 s.

U73122 (Sigma-Aldrich, Saint Louis, MO) specifically inhibits phospholipase C and A_2_, enzymes crucial for the hydrolysis of PI (phosphatidylinositol) to IP_3_ (inositol triphosphate), thereby reducing the levels of free cytosolic Ca^2+^. It also acts to inhibit the coupling of GPCR activation without influencing the production of cAMP. U73122 used as a positive control and was added to the assay for [Ca^2+^]_i_ measurement.

**PF4 ELISA analysis**

Samples were collected and the change of PF4 concentrations between different groups were detected by ELISA according to the manufacturer’s protocol (Colorful-Gene Biotech, Wuhan, China).

**UPLC analysis**

The analysis was performed on a ZORBAX SB-C18 column (4.6 mm×100 mm, 1.8 μm) from Agilent (Agilent Technologies, Santa Clara, California). The detective wavelength was set at 280 nm. The mobile phases of 0.1% formic acid-water (A) and Acetonitrile (B) are as follows: 0-7 min, 5-17% B; 7-14 min, 17-25% B; 14-16 min, 25-28% B; 16-17 min, 28-30% B; 17-22 min, 30-55% B; 22-24 min, 55-71% B; 24-27 min, 71-85% B; 27-30 min, 85-85% B. The ﬂow rate was 0.5 mL/min and the column temperature was 50°C. The specificity, precision, linearity, repeatability, and stability of the method were validated.

**Molecular docking study of SAA binding to P2Y_1_ and P2Y_12_**

The molecular docking assay was performed using the Surflex-Dock program interfaced with SYBYL X 1.3 software to dock the compounds into the binding site of P2Y_12_ and P2Y_1_. The two-dimensional (2D) structures of SAA, AZD1283 and MRS2500 were obtained from PubChem Database (http://pubchem.ncbi.nlm.nih.gov), and then converted into three-dimensional (3D) structures by CONCORD algorithm, and Total Score was Calculated as the evaluation criterion of molecular docking. Docking poses inspections and ligand-receptor interaction analysis were performed by Discovery Studio 3.5 (Accelrys). During the preparation of the protein structure, all the water molecules and co-crystallized ligands were removed. Hydrogen atoms were added to the protein, followed by energy minimization and charge calculations (AMBER7FF99). For protoMol generation, threshold of 0.5 and the bloat value of 1 were set, and the position of the co-crystallized ligand AZD1283 and MRS2500 was considered the potential binding pocket of SAA.

**Supplemental Results**


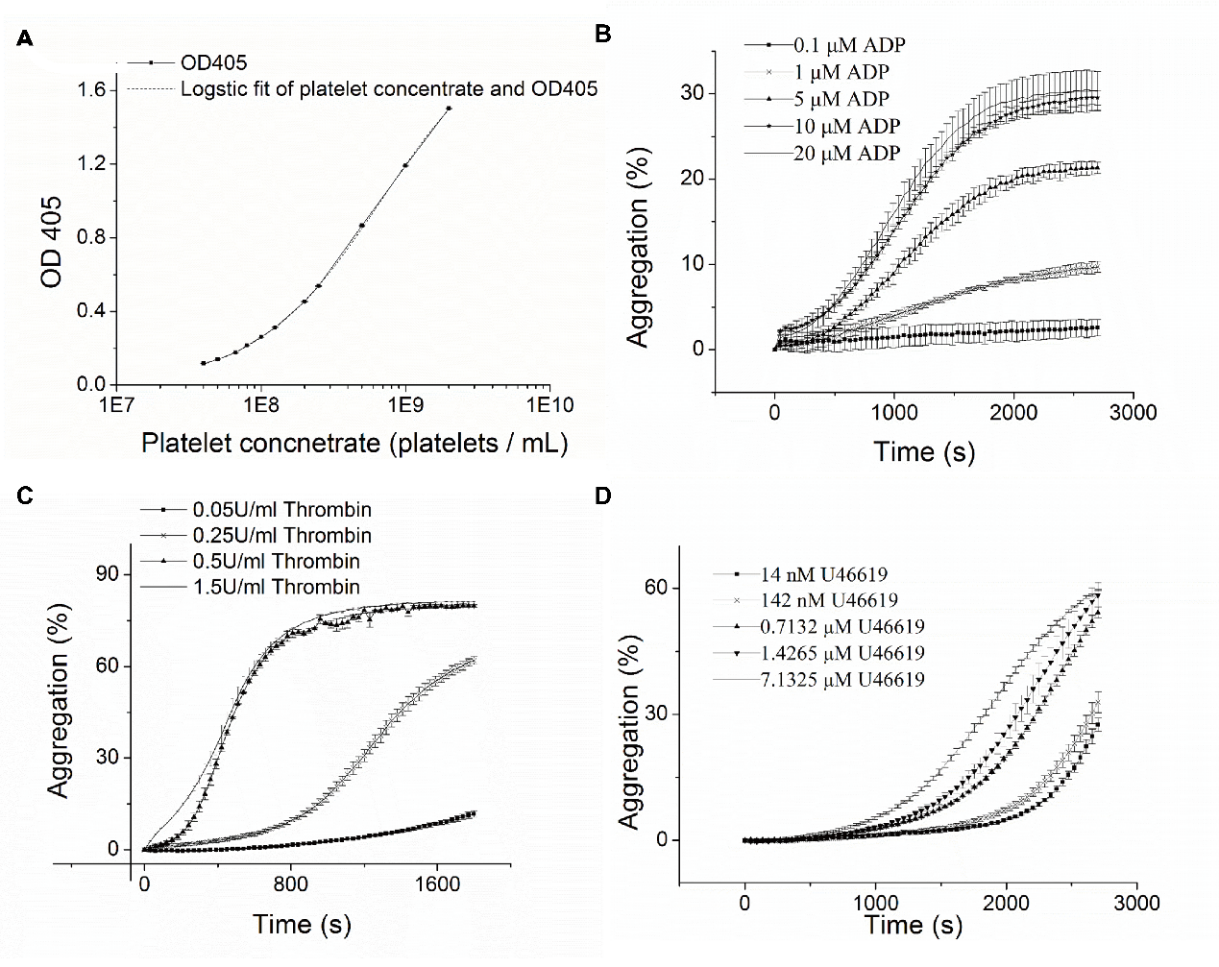


**Figure S1** (A) The coefficient between OD405 value and the concentration of platelets. (B-D) Agonists for the GPCRs (ADP, Thrombin and U46619) all triggered platelets aggregation in a dose-dependent manner (n = 3).


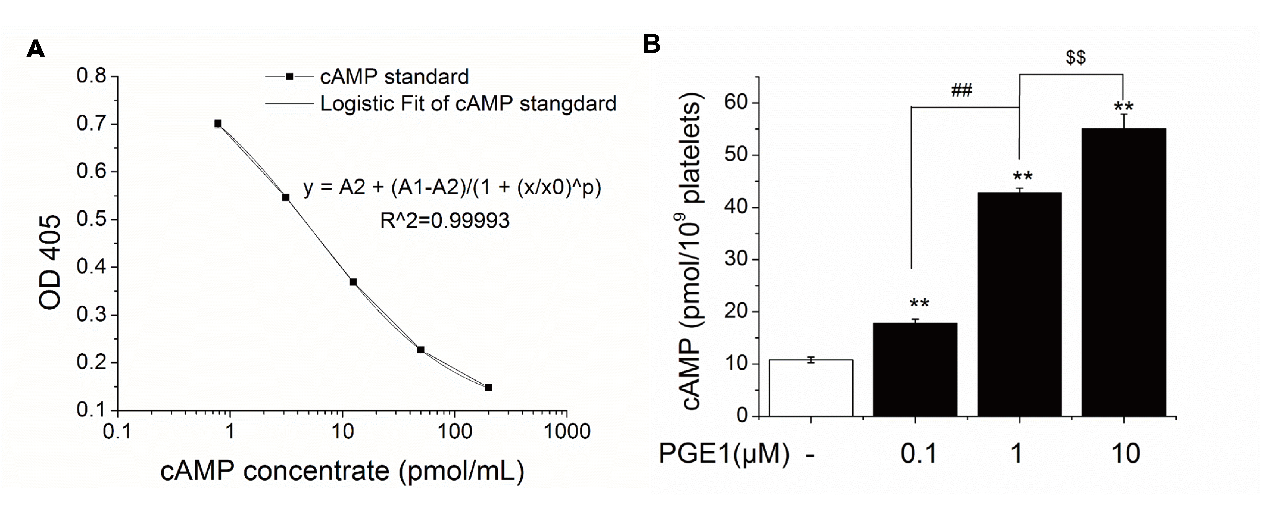


**Figure S2** (A) The correlation between the cAMP concentration and OD405 value. (B) PGE1 increased the production of cAMP in dose dependent manner (n = 3, ***p* < 0.01: platelets treated with three different concentrations of PGE1 compared to no PGE1, *^##^p* < 0.016.25: 0.1 μM PGE1 compared to 1 μM PGE1, *^$$^p* < 0.01: 1 μM PGE1 compared to 10 μM PGE1).
